# Prevalence, types, and malformations in congenital anomalies of the kidney and urinary tract in newborns: a retrospective hospital-based study

**DOI:** 10.1186/s13052-019-0635-9

**Published:** 2019-04-18

**Authors:** Zhong-yi Li, Yan-min Chen, Li-qian Qiu, Dan-qing Chen, Chong-gao Hu, Jian-yun Xu, Xiao-hui Zhang

**Affiliations:** 1 Department of Urology, The Second Affiliated Hospital, Zhejiang University School of Medicine, 88 Jiefang Road, Hangzhou, Zhejiang Province 310006 People’s Republic of China; 2 Department of Obstetrics, Women’s Hospital, Zhejiang University School of Medicine, No.1 Xueshi Road, Hangzhou, Zhejiang Province 310006 People’s Republic of China; 3 Department of Women’s Health, Women’s Hospital, Zhejiang University School of Medicine, No.1 Xueshi Road, Hangzhou, Zhejiang Province 310006 People’s Republic of China; 4 Zhejiang Provincial Center for Disease Control and Prevention, 3399 Binsheng road, Hangzhou, People’s Republic of China

**Keywords:** Congenital anomalies of the kidney and urinary tract (CAKUTs), Prevalence, Associated malformations, Prenatal detection

## Abstract

**Background:**

Congenital anomalies of the kidney and urinary tract (CAKUTs) are some of the most common birth defects affecting newborns. CAKUTs often have poor birth outcomes owing to the limited experience of physicians in developing countries regarding antenatal and postnatal diagnosis. We aimed to estimate the epidemiology of CAKUTs using data from a hospital-based registry in Zhejiang Province, China.

**Methods:**

We included a total of 2790 newborns with CAKUTs, identified among 1,748,038 births during 2010–2016. The prevalence and type of CAKUTs, maternal and neonatal characteristics, and associated malformations were analyzed.

**Results:**

The average prevalence of CAKUTs born to mothers overall and mothers aged ≥35 years were both around 1.60 per 1000 births (95% confidence interval (CI), 1.54–1.66; 95% CI, 1.44–1.83, respectively) during the study period. The prevalence of CAKUTs changed over time among all women and women of advanced maternal age, although no significant trends were observed. CAKUTs were more likely to occur in male than female newborns (odds ratio (OR) 1.28, 95% CI 1.18–1.38), in multiple births than singletons (OR 1.53, 95% CI 1.21–1.92) and in urban areas than rural areas (OR 1.27, 95% CI 1.18–1.37). The overall prenatal detection rate of CAKUTs was 73.87%. The average gestational age at antenatal diagnosis was 26.57 ± 8.70 weeks. A total 22.69% CAKUTs had associated malformations. Congenital heart defects were the most common anomalies, accounting for 8.89% of the whole population. The main proportion in subgroups was hydronephrosis, representing 31.79% of registered CAKUTs.

**Conclusions:**

There was a nearly twofold increase in the prevalence of CAKUTs from 2010 to 2016 in Zhejiang Province. CAKUTs are strongly associated with male sex, multiple births, urban areas, and other nonurinary congenital malformations.

## Background

Congenital anomalies of the kidney and urinary tract (CAKUTs) are some of the most common birth defects in newborns. CAKUTs include a broad spectrum of structural and functional abnormalities of the kidney, collecting system, bladder, and urethral abnormalities. The prevalence of CAKUTs ranges from 4.2 per 10,000 births in Taiwan to 4.0 per 1000 births in Russia and some Asian and European countries [1–5]. Over half of pregnancies involving severe CAKUTs have adverse birth outcomes, such as stillbirth or even spontaneous abortion [6]. Among live births, CAKUTs are closely associated with impaired renal function, particularly chronic kidney and kidney-related diseases in young adults [7–10]. For example, approximately 39.8% of end-stage renal disease cases in patients younger than 20 years old in Japan are owing to CAKUTs [9]. Additionally, CAKUTs are likely to be associated with malfunctions in other organs [5, 11]. Owing to improvement in antenatal diagnostic technologies, most CAKUTs can be identified and classified at an early stage of pregnancy using ultrasonography, radiation, computed tomography (CT), magnetic resonance imaging (MRI), and new genetic approaches [5, 12–15].

China has seen few comprehensive evaluations of CAKUTs in newborns. A previous study covering 264 patients showed that 43.9% of chronic kidney disease (CKD) cases in children were owing to CAKUTs [16]. Owing to the poor outcomes of CKD in children and the huge population in China, it is necessary to have an accurate estimate of CAKUTs [9, 16, 17]. Zhejiang Province is located on the eastern coast of China, where a provincial birth defect surveillance system has been established for over 30 years. This provides a valuable platform for scientific research on CAKUTs.

This study aimed to evaluate the epidemiology of CAKUTs using data from a hospital-based birth defect surveillance system in this province. To the best of our knowledge, this is the first work to give full consideration to this topic at provincial level. Our findings may be beneficial for clinical guidance and policy making.

## Methods

### Study design and data sources

This was a retrospective study based on data from a birth defect surveillance system in China’s Zhejiang Province. In this study, participants included all births and all ascertained patients with CAKUTs registered in the system from 2010 to 2016.

The birth defect surveillance system in Zhejiang Province is a hospital-based registry system covering 91 delivery hospitals. The average number of births per year recorded in the system represents about 30% of total births in this province. The registry contains information from clinical records and routine antenatal care facilities. The data include maternal characteristics, malformation subtypes, pregnancy outcomes, and newborn birth records. Here, we collected information of all births, including early fetal loss, live births, and stillbirths after at least 28 weeks of gestation. Cases with anomalies were assessed by experts within 7 days of delivery. Regardless of gestational weeks and pregnancy outcomes, all anomalies were reported online by well-trained medical staff. Quality control was strictly carried out by physicians in surveillance hospitals and the Women’s Hospital, School of Medicine Zhejiang University throughout this work.

The Committee for Research Ethics at Zhejiang Provincial Center for Disease Control and Prevention approved the study (number: 2018KY036). All information was confidential.

### Antenatal care and CAKUTs identification

In China, all pregnant women are required to make 7–11 antenatal visits during the course of their pregnancy, at gestational ages 6–13, 14–19, 20–24, 25–28, 29–32, 33–36, and 37–41 weeks. Women with pronounced risk factors, such as advanced maternal age, previous adverse pregnancy outcomes, specific complications, or abnormal family history, are encouraged to make additional prenatal visits.

Normally, maternal serum screening is recommended in the first and second trimesters. Similarly, ultrasound is offered for nuchal translucency screening at 11–13 weeks of gestation. At 20–24 weeks of gestation, systematic prenatal ultrasound screening is recommended, to confirm the presence or absence of most malformations. Noninvasive prenatal testing has been used since 2013 and is offered to eligible women at 12–22 weeks of gestation. When necessary, amniocentesis and chorionic villous sampling are usually conducted at 16–20 weeks of gestation. Radiation, CT, MRI, and genetic testing are also used to confirm anomalies. Autopsies can be helpful, but they are not widely performed.

With respect to CAKUTs screening, all fetuses undergo routine ultrasound scans. In this study, diagnoses of CAKUTs were made by obstetricians and pediatricians. CAKUTs were classified according to the International Statistical Classification of Diseases and Related Health Problems 10th Revision (ICD-10), codes Q60–Q64. Furthermore, postnatal examination was suggested for suspected prenatal anomalies. Fetuses with unconfirmed defects were not reported. Here, a diagnosis of hydronephrosis was made when the maximum diameter of the renal pelvis was ≥10 mm on a prenatal ultrasound scan after 30 weeks of gestation.

### Statistical analysis

The prevalence of CAKUTs was calculated as the number of cases per 1000 births, including early fetal loss, live birth, and fetal death at or after the 28th week of gestation. Cases with associated malformations were only counted as one case when we presented overall prevalence. However, these cases were allocated to different subgroups and counted separately.

The chi-square test was used to compare the changes in prevalence of CAKUTs among all women and among women with advanced age. Odds ratios (ORs) and 95% confidence intervals (CIs) were used to explore the associations of CAKUTs with maternal characteristics and birth outcomes. The proportion of each subgroup of CAKUTs and associated malformations were presented. All *P*-values were two-tailed and values below 0.05 were considered significant. SPSS 19.0 software (SPSS Inc., Chicago, IL, USA) was used for data analysis.

## Results

### Trends in the prevalence of CAKUTs

There were 2790 CAKUTs identified among 1,748,038 births during 2010–2016, yielding an average prevalence of 1.60 per 1000 births (95% CI, 1.54–1.66). Among births to women aged ≥35 years, the prevalence of CAKUTs was 1.63 per 1000 births (95% CI, 1.44–1.83). The prevalence of CAKUTs changed over time among all women and women with advanced age, despite no significant linear trends (Table 1).

**Table 1:** Prevalence trends of congenital anomalies of the kidney and urinary tract (CAKUTs) among total and women aged ≥35 years

Time	Born to all women			Born to women aged ≥35 years		
	Number of births	Number of cases of CAKUTs	Prevalence per 1000 births	Number of births	Number of cases of CAKUTs	Prevalence per 1000 births
2010	200,008	217	1.08	22,539	14	0.62
2011	221,469	229	1.03	19,557	18	0.92
2012	253,540	428	1.69	22,397	44	1.96
2013	266,036	432	1.62	22,627	47	2.08
2014	265,289	431	1.62	23,635	36	1.52
2015	264,029	474	1.80	26,608	52	1.95
2016	277,667	579	2.09	29,345	61	2.08
average	1,748,038	2790	1.60	166,708	272	1.63
		*x*^2^=126.57			*x*^2^=29.96	
		*P <* 0.001			*P <* 0.001	

Abbreviations: *CAKUTs* congenital anomalies of the kidney and urinary tract

### Maternal characteristics and birth outcomes in CAKUTs

Table 2 shows maternal characteristics and birth outcomes in CAKUTs. Among 2790 patients with CAKUTs, the number and proportion of males, females, and births with unclear sex were 1583 (56.73%), 1096 (39.28%), and 111 (3.98%), respectively. Males had a higher risk of CAKUTs (OR 1.28, 95% CI 1.18–1.38) than females. The sex ratio was 1.44:1. Further, multiple births and births in urban areas presented a higher risk of CAKUTs than singletons (OR 1.53, 95% CI 1.21–1.92) and births in rural areas (OR 1.27, 95% CI 1.18–1.37). CAKUTs were poorly associated with maternal age. A total 2061 cases with CAKUTs were recognized prenatally, yielding a prenatal detection rate of 73.87%. The average gestational age at antenatal diagnosis was 26.57 ± 8.70 weeks.

**Table 2:** Maternal characteristics and birth outcomes in CAKUTs

Variable	Total births	Number of cases of CAKUTs	Prevalence per 1000 births	OR 95% CI
Infant gender				
Male	917,758	1583	1.72	1.28 (1.18–1.38)*
Female	818,510	1096	1.34	Ref
Maternal age				
< 20	48,776	52	1.06	0.65 (0.50–0.86)**
20-	396,705	574	1.45	0.89 (0.80–0.99)***
25-	761,274	1243	1.63	Ref
30-	374,560	650	1.74	1.06 (0.97–1.17)
≥ 35	166,708	271	1.63	1.00 (0.87–1.14)
Multiple births	31,121	75	2.41	1.53 (1.21–1.92)*
Single birth	1,716,902	2715	1.58	Ref
Region				
Urban	875,763	1562	1.78	1.27 (1.18–1.37)*
Rural	872,275	1228	1.41	Ref

Abbreviations: *CAKUTs* congenital anomalies of the kidney and urinary tract, *OR* odds ratio, *CI* confidence interval *P<0.001 **P=0.002 ***P=0.016

### Subtypes of CAKUTs and associated malformations

Overall, 22.69% of births with CAKUTs had associated malformations; in male and female newborns, the corresponding proportion was 20.34 and 21.90%, respectively. The most common coexisting anomalies were congenital heart defects, accounting for 8.89% of all registered cases. Most associated malformations were reported more frequently in male than in female newborns.

The most frequent CAKUT was hydronephrosis, representing 31.79% of all CAKUTs. This was followed by other CAKUTs, polycystic kidney, renal agenesis, renal ectopia, and renal duplication (Table 3).

**Table 3:** Associated anomalies and distribution of CAKUTs, by sex

Subtype	Total (2790)		Male (1583)		Female (1096)		Male/ Female
	n	%	n	%	n	%	
Associated anomalies	633	22.69	322	20.34	240	21.90	1.34
Subtypes							
Congenital heart defects	248	8.89	129	8.15	97	8.85	1.33
Limb defects	56	2.01	30	1.90	17	1.55	1.76
Neural tube defect	19	0.68	7	0.44	7	0.64	1.00
Hydrocephalus	16	0.57	7	0.44	6	0.55	1.17
Trisomy 21	6	0.22	6	0.38	0	0.00	–
Pcromphalus	9	0.32	7	0.44	1	0.09	7.00
Diaphragmatocel	5	0.18	3	0.19	1	0.09	3.00
Cheilopalatognathus	16	0.57	5	0.32	9	0.82	0.56
Other	368	13.19	176	11.12	141	12.86	1.25
Subtypes of CAKUTs							
Hydronephrosis	890	31.90	666	42.07	207	18.89	3.22
Polycystic kidney	533	19.10	245	15.48	258	23.54	0.95
Renal agenesis	529	18.96	241	15.22	270	24.64	0.89
Renal ectopia	121	4.34	59	3.73	61	5.57	0.97
Renal duplication	67	2.40	32	2.02	35	3.19	0.91
Others	678	24.30	349	22.05	281	25.64	1.24

Abbreviation: *CAKUTs* congenital anomalies of the kidney and urinary tract

## Discussion

In this study, the prevalence of CAKUTs was 1.60 per 1000 births, with 95% CI 1.54–1.66. This rate is much lower than in reports from Copenhagen University Hospital, Murmansk County in Russia, and western areas of Saudi Arabia, where the corresponding data were around 3–4 cases per 1000 births; however, our finding is far higher than that in Taiwan (4.2 per 10,000 births), and similar to that in Hunan, China (11.7 per 10,000 births) [1–4, 18, 19]. Such inconsistencies could be explained by differences in sociodemographic background, malformation inclusion criteria, study population, diagnostic technology and surveillance quality. In Murmansk County, a population-based birth registry recorded anomalies diagnosed from 22 weeks of gestation to hospital discharge [1]. Those authors admitted that some figures had been overestimated owing to the lack of strict diagnostic criteria for pyelectasis, hydronephrosis, and unspecified anomalies [1]. In Saudi Arabia, the high rate of consanguineous marriage within the local population might increase the rate of CAKUTs [2]. The study in Denmark followed up a birth cohort for 8 years [3]. In our study, the prevalence might be underestimated because all anomalies were restricted to within 7 days after birth, similar to the study in Taiwan [4]. Longitudinal postnatal follow-up is recommended, as some malformations might be revealed later in life. Finally, this wide interval of CAKUTs prevalence could be owing to the currently limited literature.

In our study, the prevalence of CAKUTs doubled from 2010 to 2016. Rising rates of CAKUTs have also been observed in parts of Europe and in Korea but were not reported in studies performed in Murmansk County of Russia and Taiwan [1, 4, 20–23]. The increasing prevalence might be owing to increased screening, developments in ultrasound technology, and improved birth defect surveillance in our study. The upward trend in prevalence of CAKUTs in Europe agrees with this view [22, 23]. The underlying cause of CAKUTs and related risk factors should be fully considered to reflect the true increase in prevalence. To our knowledge, CAKUTs are caused by genetic modifications in the kidney that are related to development, and as a result of exposure to drugs or fetal environmental factors, as indicated in mouse models and models of developmental diseases in humans [14, 24, 25]. Maternal obesity, fertility treatments, multiparity, infectious diseases, and diabetes during pregnancy could increase the occurrence of CAKUTs [1, 4, 26, 27]. In 2014, the Chinese government initiated a policy encouraging people to have a second child. The proportion of pregnant women with advanced age and who have complications has been on rise. This might partly explain the increase in the rate of CAKUTs found in our study.

In the present study, a high proportion of CAKUTs was reported in male newborns, newborns from multiple births, and newborns from urban areas. A population-based case-control study in Taiwan showed that males had a 1.83-fold greater risk of CAKUTs than females [4]. A clear male predominance in CAKUTs has been documented in numerous studies. The sex ratio of CAKUTs is around 2:1–3:1, which includes patients who undergo postnatal follow-up [2, 7]. In reports from Saudi Arabia, about 66% of cases were male; however, it excluded cases of CAKUTs at less than 20 gestational weeks [2]. In our study, the sex in about 4% of CAKUTs was unknown because we included early fetal loss. This proportion was larger than the corresponding data in Taiwan and Saudi Arabia (less than or around 2%) [2, 4]. Nevertheless, the sex ratio of CAKUTs in our study was comparable to a national survey of children with end-stage renal disease in China [17] Sex-specific mechanisms should be investigated with respect to environmental stress, the renal transcriptome, and gene expression and pathways.

A rising rate of multiple births in Zhejiang Province has been previously reported [28]. With socioeconomic development, delaying childbirth has become popular among women in China. The increasing proportion of women with advanced reproductive age has been strongly linked to the increasing use of assisted reproductive technology. Both fertility treatments and multiple births might increase the risk of birth defects, including urogenital system abnormalities [29–31]. In our study, the prevalence of CAKUTs differed obviously by region. Similarly, urban areas of China showed higher prevalence of congenital heart defects in Hunan, and higher overall prevalence of birth defects in Dalian city [19, 32]. This could be attributed to greater awareness of health care among women in urban areas, which promotes better utilization of antenatal care. Likewise, women residing in urban areas face greater risks from environmental exposures that could lead to malformations. We found poor associations between maternal age and CAKUTs, which was supported by the Murmansk County Birth Registry Study [1]. However, in Taiwan, advanced maternal age increased the risk of CAKUTs [4]. Nevertheless, among nonchromosomal anomalies, maternal age was also linked with CAKUTs to some extent, but with a smaller effect than chromosomal anomalies [21]. The proportion of CAKUTs associated with chromosomal anomalies was also lower in our study.

It has been reported that 6–50% of CAKUTs are associated with malformations [1–3, 11, 25, 26]. The proportion of non-isolated anomalies in our study was relatively low, which could be owing to a relatively low prevalence of CAKUTs. Among 1678 CAKUT cases in northeastern France with 2 years’ follow-up, 34% had associated malformations [11]. Variability in the length of follow-up and study population should be taken into account. Our results showed congenital heart defects to be the most common anomalies associated with CAKUTs, followed by abnormalities of the limbs. Congenital heart defects are the leading malformations in Zhejiang Province. It has also been reported that CAKUTs are one of the most common defects associated with congenital heart defects [33, 34]. According to published studies, the most frequently reported nonurinary anomalies involve the genitourinary tract; central nervous system, musculoskeletal, digestive and cardiovascular systems, and chromosomal anomalies [2, 5, 11].

In our study, the most frequently detected subgroup of CAKUTs was hydronephrosis, similar to most reports [1–3, 11]. However, the distribution of hydronephrosis showed some differences across studies. In Saudi Arabia, over 50% of CAKUTs were hydronephrosis; in northeastern France and Murmansk County, hydronephrosis accounted for 28.9 and 14.3% of CAKUTs, respectively [1, 2, 11]. These results depend largely on variation in the diagnostic criteria and study population. As noted, in the study from Saudi Arabia, mild hydronephrosis was defined as maximum diameter 5–10 mm whereas in Murmansk, multiple births were excluded [1, 2]. In those reports, unspecified malformations were less commonly reported because some cases were not confirmed after discharge; this proportion was lower in our study than in Murmansk, where half of malformations were other CAKUTs [1]. The prenatal detection rate of CAKUTs in our study was higher than that recorded in the Murmansk County Birth Registry Study (42.1%), similar to the rate in northeastern of France (71%), but lower than the level in the Netherlands (over 80%) [11, 22]. In the Netherlands, this was mainly attributable to a large proportion of anomalies of the collecting system (70.4%) and high coverage of prenatal screening. The average term at prenatal diagnosis in our study was similar to the study in France (26.6 ± 9.2 weeks) [5].

A strength of the current work is that we used provincial data, to provide a more thorough understanding of the epidemiology of CAKUTs. The most important strength of this study is the large sample size covering over 1.5 million births, which yielded reliable results. However, the potential limitations of this work should also be mentioned. First, the diagnosis of CAKUTs was confirmed within 7 days of birth but before the child was discharged from the hospital. This may have had some negative influence on comparisons with developed countries that maintain universal population-based registries and conduct long-term observation. Second, misclassification can not be disregarded in the higher proportion of unspecified anomalies, accounting for 24.3% of CAKUTs. Lastly, there is a likelihood of underreported risk factors related to birth defects, such as maternal smoking and environmental exposure during pregnancy, owing to limited data [35, 36].

## Conclusion

In conclusion, despite a relative low prevalence of CAKUTs in our study, the rising trend in prevalence merits further attention. Routine screening of CAKUTs should be performed to identify congenital malformations, particularly abnormalities of the cardiovascular system. Once the diagnosis of CAKUTs is made, a complete assessment of coexisting anomalies and suggestions for postnatal care should be provided.

## Abbreviations

CAKUTs: Congenital anomalies of the kidney and urinary tract
CI: Confidence interval
CT: Computed tomography
ICD: International Statistical Classification of Diseases
MRI: Magnetic resonance imaging
OR: Odds ratio
